# Novel Nanomaterials for Developing Bone Scaffolds and Tissue Regeneration

**DOI:** 10.3390/nano15151198

**Published:** 2025-08-05

**Authors:** Nazim Uddin Emon, Lu Zhang, Shelby Dawn Osborne, Mark Allen Lanoue, Yan Huang, Z. Ryan Tian

**Affiliations:** 1 Cell & Molecular Biology, University of Arkansas, Fayetteville, AR 72701, USA; 2 Institute for Nanoscience & Engineering, University of Arkansas, Fayetteville, AR 72701, USA; 3 Environmental Dynamics, University of Arkansas, Fayetteville, AR 72701, USA; 4 Animal Science, Division of Agriculture, University of Arkansas, Fayetteville, AR 72701, USA; 5 Chemistry and Biochemistry, University of Arkansas, Fayetteville, AR 72701, USA

**Keywords:** inorganic nanomaterials, synthetic polymers, bone scaffold, signaling pathways, regulatory issues, tissue regeneration

## Abstract

Nanotechnologies bring a rapid paradigm shift in hard and soft bone tissue regeneration (BTR) through unprecedented control over the nanoscale structures and chemistry of biocompatible materials to regenerate the intricate architecture and functional adaptability of bone. This review focuses on the transformative analyses and prospects of current and next-generation nanomaterials in designing bioactive bone scaffolds, emphasizing hierarchical architecture, mechanical resilience, and regenerative precision. Mainly, this review elucidated the innovative findings, new capabilities, unmet challenges, and possible future opportunities associated with biocompatible inorganic ceramics (e.g., phosphates, metallic oxides) and the United States Food and Drug Administration (USFDA) approved synthetic polymers, including their nanoscale structures. Furthermore, this review demonstrates the newly available approaches for achieving customized standard porosity, mechanical strengths, and accelerated bioactivity to construct an optimized nanomaterial-oriented scaffold. Numerous strategies including three-dimensional bioprinting, electro-spinning techniques and meticulous nanomaterials (NMs) fabrication are well established to achieve radical scientific precision in BTR engineering. The contemporary research is unceasingly decoding the pathways for spatial and temporal release of osteoinductive agents to enhance targeted therapy and prompt healing processes. Additionally, successful material design and integration of an osteoinductive and osteoconductive agents with the blend of contemporary technologies will bring radical success in this field. Furthermore, machine learning (ML) and artificial intelligence (AI) can further decode the current complexities of material design for BTR, notwithstanding the fact that these methods call for an in-depth understanding of bone composition, relationships and impacts on biochemical processes, distribution of stem cells on the matrix, and functionalization strategies of NMs for better scaffold development. Overall, this review integrated important technological progress with ethical considerations, aiming for a future where nanotechnology-facilitated bone regeneration is boosted by enhanced functionality, safety, inclusivity, and long-term environmental responsibility. Therefore, the assimilation of a specialized research design, while upholding ethical standards, will elucidate the challenge and questions we are presently encountering.

## 1. Introduction

Bone damage, even a minute crack or fracture, can encompasses a painful long path of self-renewal repairing process [1,2]. The most common occurrence related to bone regeneration has been witnessed in bone fracture infirmities. In fact, in order to fix even minor bone tissue damage and fractures, the self-healing process involves systematic cell (e.g., osteoblasts, osteocytes and osteoclasts), biomineral, and hormone regeneration [3]. In more severe circumstances, for instance, while removing tumors, fixing traumatic bone damage and restoring the missing bone tissues, mammals require invasive surgeries to complete the healing process. Inter alia, the current surgical process presents significant challenges in the basic, translational, and clinical fields of orthopedic treatment [4]. Again, the consequence of a poor fixation may defer the fracture fixation [5]. Hence, restoring bone tissue through major surgical intervention such as autograft or allograft methods often lead to more invasive bone transplant surgery. These surgeries do not merely trigger painful complications but also increase the risk of deadly transmitting infections and post-traumatic complications. Allografting creates a high chance of transplanting numerous biological components, such as immune cells, fibroblasts, bone cells and matrix from the donor [6]. Furthermore, immune rejections are notable considerations to be measured in bone transplants and autografting, which must be addressed to avoid the triggering of autoimmune diseases [2].

A longstanding challenge in bone regeneration is the attainment of graft-versus-host disease (GVHD), where the recipient’s immune system attacks and then rejects the implanted bone scaffolds. In this case, the patient has to be treated with immunosuppressive therapy before and after the surgery [7]. Major post-treatment risks that nanomedicine can potentially help minimize include secondary hemorrhage, bacterial colonization, inflammation and sensory disturbance among others [8]. A common approach is autograft, which involves harvesting bone tissue via major surgery from elsewhere in the patient’s body for transplantation to the bone-damage location via another surgery within a short period of time. This procedure is meant to minimize the immune system attacking the transplanted tissues, however, this procedure can bring in potential risks of infection, severe pain and possible nerve damage [6]. Additional surgery to harvest a patient’s tissue often results in prolonged recovery time. A further concern is that if the gap within the fracture is too wide for new blood vessels to span, the new tissue cannot grow naturally due to the restriction of nutrients and oxygen which becomes a prohibitive factor for rapid regeneration [9]. Therefore, conservative bone grafting with alternative nanotechnologies can be used to address the complex challenges associated with bone tissue regeneration.

The upsurging problems associated with impaired bone treatment, costs in the current medical intervention, and limitations regarding bone grafting or implanting sheds light on the need for an efficient breakthrough in tissue engineering (BTE) for maximizing bone regeneration. The processes of natural bone formation, embryonic development, and fracture healing mechanisms provide us valuable keys, insights, and motivations for the development of new strategies to combat the limitations in BTR. Countering the troublesome issues related to bone autograft and the concurrent advancement in BTE has paved the way for a solution in the form of a three-dimensional (3D) artificial bio-scaffold. Primarily, 3D artificial bio-scaffolds are comprised of a bone-like structure and stem cell-embeddable extracellular matrices (ECM) [10]. A notable aspect of these bio-scaffolds is their interconnecting porous structure, which is designed to serve as a physical template for cell adhesion, development, and proliferation. The interconnecting template of a bone scaffold is structured with organic and inorganic composites. Analogous to other tissue types, bone tissues are supported by an ECM which grips essential materials for cell sustenance [11]. Briefly, the ECM of the scaffold is an independent complex matrix that is concreated with a combination of inorganic and organic components [11,12]. The organic structure consists of a framework of crosslinked collagen (a protein) fibrils that self-assemble into fibers. Theoretically, one of the primary interests in bone tissue engineering is the development of a new osteogenic scaffold through the growing, differentiating, and proliferating bone marrow-derived mesenchymal stromal cells (MSCs), adult progenitor osteogenic cells (POCs), or hematopoietic stem cells (HSCs) in an ECM matrix. Here, the inclusion of supportive growth factors (GFs) and nutrients supports the growth and development of stem cells [13,14]. Next, the multipotent stem cells transform into the osteoblasts, adipocytes and chondrocytes upon proper stimulation. BTR typically uses multipotent MSCs which express CD75, CD90, and CD105 on their surface [15]. Furthermore, optimizing the ECM’s biological and mechanical properties, ensures durable functionality and stability and can pose significant technological challenges. Conversely, the regeneration techniques must ensure the presence of essential biomolecules, GFs, biominerals, mechanical support, and bio-signals propagation [16]. These elements are essential for facilitating optimal metabolism and communication between cells. Combining these concepts, the formulation of an artificial bone bio-scaffold is a notable progression in biomedical engineering and molecular biology, where a scaffold is designed meticulously to develop a template for BTR and guide the formation of new bone with the desired size and shape [17]. However, the goal of these bio-scaffolds is to regenerate injured bone tissue by interacting with the surrounding biological fluids. Hypothetically, an ideal synthetic bone scaffold should possess a physiologic and reliable biological environment that enhances osteogenic conduction and induction to promote osteogenesis [18].

In addition, the development of advanced nanotechnology has facilitated the incorporation of nanoscale materials into bone tissue engineering. Nanostructured materials such as nanospheres, nanowires, nanotubes, nanofibers, nanorods, quantum dots, and nanosheets exert unique and ideal characteristics, including structural, biological, mechanical, and functional properties for bone tissue engineering. Their distinct structures and surface chemistry enable them to replicate the complex three-dimensional architecture of bone effectively [19]. These materials possess the ability of osteogenic conduction and osteogenic induction because several NMs can craft a conducive environment for the bone tissue [20]. Their functionality encompasses labeling and targeting cells within the scaffolds. Additionally, particular NMs provide postoperative tracking and analysis owing to their ability to possess magnetic or electromagnetic properties. For the successful application of nanoparticles, this is indispensable as it ensures colloidal stability within the dispersion medium and prevents the aggregation of the NMs [21]. When designing a bio-scaffold with such nanomaterials for biomedical applications, the biocompatibility, safety, and absence of intrinsic cytotoxicity emerge as the most critical issues that must be taken into consideration [22] given that implant-induced infections are the fundamental challenge associated with grafting. It is noted that some NMs can serve as a source of antibacterial agent.

This review covers contemporary findings, insights, innovations, and recommendations on designing scaffolds using NMs of calcium phosphate derivative, metallic oxides, and FDA-approved synthetic polymers. Upon careful consideration of previous research in this field, we observed that none have provided in-depth information regarding the design of a scaffold using the aforementioned materials. There have been numerous studies conducted on this topic, but none of them have provided adequate guidelines for the design, implementation, or limitations of the NMs. To tackle the obstructions and enhance scientific understanding in scaffold technology, we conducted this critical analysis and endorsed concurrent research aimed at resolving existing challenges and establishing future trajectories in BTR. In detail, this review demonstrates current gaps and unmet challenges regarding NMs-based scaffold production. Furthermore, we will critically emphasize the modern manufacturing techniques available and accessible that can address the constraints behind achieving angiogenesis, enduring mechanical stability, biocompatibility, and cellular sustainability for effective bone tissue regeneration.

## 2. Material Issues in Regenerating Bone Tissue

Bone tissue regeneration involves the synchronization of materials with the cell-growing environment for building an effective extracellular matrix (ECM). The ECM provides a feasible plot for stem cell seeding and for incorporating essential growth and regulatory factors to promote biomineralization, cell and tissue adhesion, growth, proliferation, differentiation, and regeneration of the bone scaffold [23]. The following sections will highlight the critical parameters related to the selection of advanced nanomaterials, which have been shown to enhance bone scaffolding.

### 2.1. Criteria for NM-Structured Scaffolding

A wide range of nanomaterials and polymers have been investigated over the decades due to their proven aptitude in bone regeneration. Empirical results from these studies promoted the integration of NMs in advanced bone scaffold designing. The unique features of the NMs offer a potential biomimicry in context to functional bone substitutes. While diameters over 100 nm and below 10 nm may entail risks of embolism and phagocytosis, leading to excretion via the spleen and kidneys, these limitations may be mitigated by precise design and advanced NM fabrication techniques [24]. Nanopatterning and nanocomposite-based nano-scaffolds are often-sought approaches for scaffold design and fabrication. The use of NMs in nanopatterning and scaffold formation imparts rapturous benefits because of their unique structural and surface features, making them versatile in bone tissue engineering.

The nanopatterning technique aims to construct synthetic ECMs for growing and developing stem cells. Controlling the surface topology of nanopatterning scaffolds can enable control over the cell behavior and influence the tissue regeneration. Moreover, their patterns are modified to create special microenvironments to spatially guide the cell growth and 3D organization to mimic the bone structure [25]. In tandem with nanoparticles, nanofibers or nanotubes as a part of NMs can mimic the fibrous structure of the ECM to provide extra support for cell adhesion and growth. The high surface-area-to-volume ratio of the nanofibers mediates cell-material interactions with the fibrous scaffold. Additionally, fibers can be fabricated using a wide range of materials, thus affording multifunctionality in the scaffold [26]. Electrospinning, phase separation and self-assembly are the most popular techniques for fabricating nanofibers in the lab regarding bone tissue engineering. Specifically, electrospinning is widely studied for fabricating fibrous NMs. Bi-, tri- and multi-axial electrospinning are often-used techniques for the creation of effectively porous and functional fibers for the BTR [27]. Lastly, nanocomposites used in BTR are composed of a combination of at least two separate components or phases, one of which has a nanoscale dimension. Because of the synergistic effects of the combination of components or phases, the final nanocomposite possesses properties that are either entirely different from or an improvement over those of its individual components alone [28,29]. Compared to the single nanomaterial, nanocomposites impart diverse multifunctionality to the scaffold that eases the efficiency and effectiveness while mimicking a natural ECM. In parallel, the meticulous design of nanomaterials requires careful consideration when working on the design of a bone scaffold. In particular, the selected nanomaterials need to have biocompatibility and require cooperation between the components for precise nascent bone formation. In addition, they must not exert any debris and barriers against the generations of new blood vessels which actively support biomolecule, oxygen, and nutrient transportation [30,31]. In addition, a nanomaterial’s bioresorbability is another fundamental criterion for scaffold development [32], because elected NMs must be degraded in vivo biological fluid and leave space for the new tissue regeneration. The degradation behavior varies depending on their intentional application. For instance, scaffold degrades after 3 to 6 months in craniomaxillofacial and 9 or more months in spinal fusion [30]. In addition, pore size and interconnectivity are critical properties that must be taken into consideration when designing a reliable scaffold. Pores in the scaffold allow the migration and diffusion of oxygen and nutrients to allow cells and tissues to grow and survive effectively, e.g., microporous scaffold walls made with nanowires allow efficient drug release into the scaffold [33,34]. The pores in the scaffold should be interconnected and the pore size should be at least 100 µm in diameter but sizes ranging from 50 to 600 µm are considered standard for the BTR application [30,35]. Recent studies claimed that multi-scale porous scaffolds consisting of macro- and micro-porosity provide better efficacy for healing and replacement compared to those using merely macroporous scaffolds. A potential drawback in having an overly porous scaffold is that the mechanical strength of the scaffold could be compromised. The meticulous design and use of particular nanocomposites for constructing porous scaffolds can overcome the drawback by introducing the ideal mechanical strength and bioactivities. Again, mechanical properties, including the strength, stiffness, toughness, and resilience properties of the scaffold, must be good enough to bear and transfer the load. The mechanical characteristics of a good scaffold must match with the host bone properties. From cortical to cancellous bone, the mechanical properties vary widely, but scaffolds must resist the mechanical impact. For instance, the cortical bone has an elevated compression strength of 100–200 MPa, whereas cancellous bone has 2–20 MPa. In addition, Young’s modulus for the cortical bone is 15–20 GPa, whereas it is 0.1–2 GPa for the cancellous bone [31,36,37]. Resisting crack propagation and withstanding repeated loading cycles are critical for durable implant stability. However, fusing the above criteria into one scaffold might be challenging and we outlined the physical and functional criteria of NMs for the scaffold formation in Table 1.

A scaffold with pores as large as 100–600 µm in size and a high porosity (≥50%) catalyzes the vascularization and osteoconductivity. Importantly, the successful passage of GFs/drugs/biomolecules is dependent on the pore size across the scaffold. Conversely, high porosity hinders the MPa and GPa passage rates. For instance, porosity greater than 70% promotes BTR but causes decreased mechanical strength. Also, enhancement of the surface area provides sites for interaction with the fluid and macromolecule interface that influence the biodegradability of the scaffold. A larger surface area creates more sites for interaction with the body fluid and macromolecule interface, thereby providing a higher rate of enzymatic and hydrolytic degradation. Alternatively, the biocompatibility of NMs in a scaffold depends on the ability of an NM to favor tissue regeneration and functional biocompatibility without exerting toxicity in a biological environment. Controlled concentration, surface area, and purity of NMs provide a favorable outcome for biocompatibility.

### 2.2. Schematic Design and Materials

**Figure 1:**
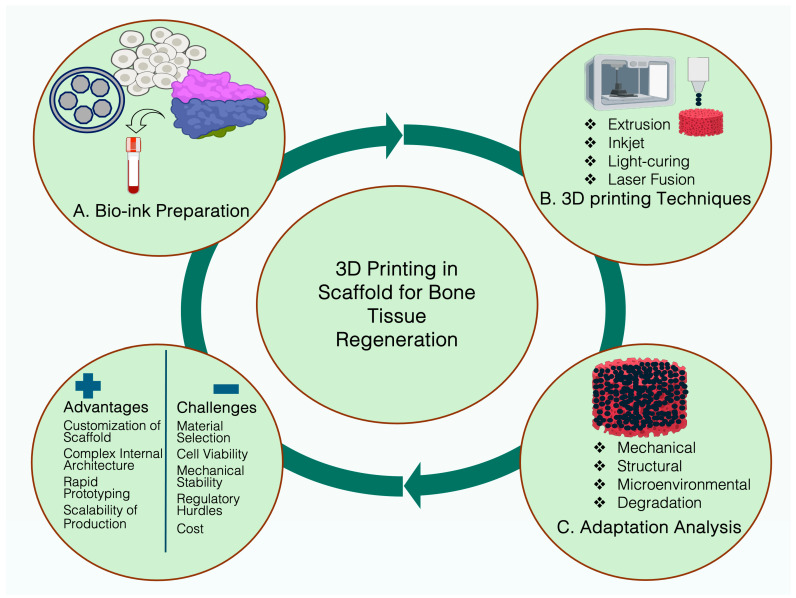
Common fabrication and adaptation techniques of 3D bioprinting.

**Figure 2:**
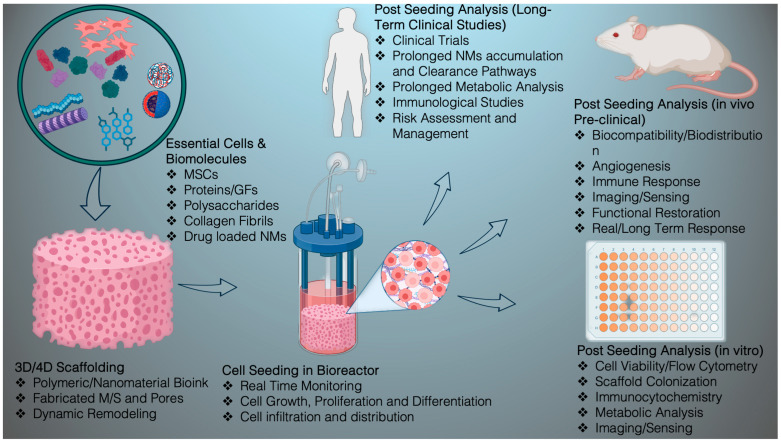
Illustration of scaffold design and preliminary parameters for in vitro, in vivo, and clinical trials in bone tissue engineering.

While designing a bone scaffold for the regeneration of defective bone, a primary concern is cell interaction and the creation of a microenvironment that can resemble bone structure or support bone tissue effectively. Secondly, the selected materials must be capable of combating immunogenicity. In addition, the NMs should decompose parallelly while the new bones are growing. Initially, the three-dimensional model has to be verified using imaging techniques and fabricated utilizing electrospinning, freeze-drying, or sol-gel techniques in a 3D/4D printer. Mainly, 3D printing enables a customized scaffold design through a layer-by-layer assembly, allowing for accurate spatial regulation of the incorporated cell populations, biomaterials, and growth factors. In tandem, 4D printing amplifies the ability of traditional 3D-printed scaffold by integrating stimuli-responsive material, thereby enabling morphological transformation over time and mimicking the natural bone scaffolds [78,79,80]. Extrusion, inkjet, and light-curing methods are popular 3D bioprinting manufacturing processes and widely used in BTR for scaffold printing. All the individual methods have their own benefits and limitations and material requirements. Briefly, the extrusion process shaped the bioinks by forcing them into a particular shape. Inkjets precisely propel the bioink onto a surface to generate outlines for designing the scaffold. Light curing often uses ultraviolet lights to transform a semi-solid or liquid material into a solid structure. In terms of NM fabrication, the electrospinning creates a nanofiber-based scaffold and freeze-drying removes the solvent from the printing materials and forms a fluffy texture. On the other hand, the sol-gel method for structuring a jelly printing material can let the gel become a solid structure by design. The produced structure contains a precise structure with a porous (>50%; size ~ 50–600 μm) and interconnected framework to facilitate cellular movement, attachment, growth, and differentiation [81]. The pores also allow nutrient transport and vascularization into the scaffold. Initially, the scaffold imitates the foundation and function of the extracellular matrix of the biomolecules. A template can provide the mechanical stability for supporting the osteogenesis. The template can be functionalized with peptides, collagen, polysaccharides, minerals, therapeutic agents and other growth factors (GFs) to create a proper environment for the stem cell seeding [82]. To increase the bioactivity and osteogenesis, the scaffold can be functionalized or coated with the bioactive molecules [83]. Then, the stem cell-seeded scaffold can be transferred into a bioreactor to provide the structural, fluidic and/or biochemical stimulations, where compression, shearing, or perfusion bioreactor triggers bone tissue maturation and mineral deposition in ECM [84]. A successful scaffold requires undergoing mechanical, structural, microenvironmental, and degradation adaptation tests. Next, the in vitro and in vivo studies can evaluate the efficacy of the scaffold. Figure 1 presents the common fabrication methods used in 3D printing and Figure 2 represents the schematic design of a scaffold, but the biological integration of various nano and biomaterials will be contingent upon the specifics of the rational design. Throughout the process, the role of NMs for reinforcing the quality of the scaffold for tissue regeneration is inevitable.

In addition, the nanomaterials have unique surface chemistry features for scaffold surface modification. The topological pattern of the nanoparticle-based surface modification is crucial for cell adhesion, growth and differentiation into the osteoblast [85]. The NMs can help to deliver osteogenic factors containing plasmids to better the osteoblast formation. Cell surface functionalization with the magnetic nanoparticle can promote the cell trafficking and guide the cells to the needy location [86]. Again, the NMs can be functionalized with the growth factors and embedded in the scaffold to accelerate the sustained release of GFs and promote osteoconductivity and osteoinductivity [87]. Certain nanoparticles are responsive to environmental stimuli by releasing, e.g., the GFs in response to alterations of the mechanical stress, pH, and temperature [88]. Even in bioreactors, nanomaterials play a substantial role. When mixed with the growth media, the nanomaterials can provide a constant supply of nutrients and GFs to boost cell growth in the bioreactor. Furthermore, nanosensors embedded in the scaffolds placed into the bioreactor can update the real-time conditions in tissue growth and degradation during bioreactor culturing. Enhancing bioavailability level is another positive aspect of nanoparticles that can increase the bioavailability of GFs and transport them to the bone growth site. In terms of the in vivo regeneration, NM-coated scaffold implants enhance the integration and acceptance of the integration into the host tissue and reduce the risk of immune rejection. Certain nanoparticles work as antimicrobial agents to prevent infections. Nanoparticles can enhance the contrast in imaging the post-implanted scaffolds and update the real-time bone regeneration process.

However, comprehensive experiments including microporous topology, mechanical strength, and biocompatibility of the chosen nanomaterials and stability as well as integrity of the scaffold in complex biological environments are required to overreach the objective. Besides, the osteoconductive potential of the scaffold can be confirmed with the RT-qPCR of osteogenic gene expression. Concurrently, the uniform distribution of the cell throughout the scaffold matrix could be verified using a confocal or optical microscope. In addition, a histological evaluation could be employed to measure tissue integration, vascularization, and maturity of tissue. Quantitative micro-CT reveals the insights into the new bone thickness.

### 2.3. Influence of Nanomaterials in Angiogenesis and Osteogenesis

Angiogenesis is an essential phase of skeletal and bone reformation since it plays a crucial role in transporting essential nutrients, oxygen, and GFs for cell survival and communication in BTR. Crosstalk in angiogenesis and osteogenic coupling is an intricate process since bone is a dynamic tissue consisting of intricate vascular networks. Therefore, several reports claimed a correlation between those processes. The development of new blood vessels allocates essential supplements to the intensively metabolically functional regenerative callus for accelerating efficient osteogenesis. Nascent blood vessels can form a functional vascular network within the scaffold for bone precursors, inflammatory cells, and cartilage to reach the injury sites [89]. Endothelial cells along with the stromal and inflammatory cells release GFs, including VEGFs, to regulate angiogenesis [90]. VEGF isomers (A-C) not only regulate angiogenesis but also form capillary-like tubes for circulation and recruiting of the endothelial progenitor cells [91]. In BTE, VEGF stimulates MSCs for differentiation and formation of bone matrix; in turn, MSCs release angiogenic factors such as fibroblast growth factor (FGF) and platelet-derived growth factors [24].

Moreover, regulating MSCs is one of the key factors for osteogenesis and many approaches have been undertaken to activate the MSCs for differentiation, proliferation, and conversion into the osteocytes. In the journey of the osteogenic process, the upregulation of transforming growth factors (TGFs), bone morphogenic protein (BMP), and runt-related transcription factors (Runx) are well recognized for deciding the differentiation and fate of MSCs. BMP family GFs belong to the TGF superfamily and they are osteoconductive; the Runx family (especially Runx-2, also known as CBFA1) is a master regulator of MSCs differentiation and TGF (especially TGF-β) signaling influences the promotion or inhibition of MSCs differentiation [92,93,94]. For instance, the integration of GFs and supplements in the MSCs growth media, hydrostatic pressure, pulsed electromagnetic fields and external and internal stimuli can facilitate osteogenic differentiation. In addition, subjunction of drug molecules such as dexamethasone to the MSCs media can trigger osteogenesis through the upregulation of runt-related transcription factors Runx-2 and osterix (OSX). As a result of the inclusion of biomolecules like β-glycerophosphate and ascorbic acids, it can trigger the secretion of type 1 collagen and accelerate osteogenesis [95]. Moreover, we are strongly hopeful of designing bone tissue scaffolds. Still, these types of materials are required to promote the formation of new blood vessels due to the intense demand for blood in the bone tissue so that they can carry nutrients and supplements [24]. Many pathways are open for nanomaterials to achieve osteo-conduction and osteo-induction efficiently. Numerous nanomaterials are already in use or under investigation in osteogenesis, especially ECM. They have reported strong functional abilities to form scaffolds with improved cell growth differentiation and proliferation efficiency for mimicking the extrinsic and intrinsic pathways of osteocyte mobility and differentiation [96]. In addition, drug or bioactive compound delivery has marked new milestones for efficient osteoblast mitigation and successfully contributed to angiogenesis as well as osteogenesis. Nanomaterials, including lipids, polymers, inorganics, and metal-based drug delivery systems, are often one-size-fits-all types of solutions for delivering drugs at a specific site via controlling TGFs, BMP, and Runx pathways [87]. Primarily, nanomaterials-based stimulation follows some particular signaling pathways, including BMP/TGF-/β, Smad2/3, MAPK/ERK, CaSR-JAK2/STAT, Wnt/β-Catenin, NF-κB, HIF-1α/VPI3K/Akt and PI3K/Akt, to regulate the angiogenesis and osteogenesis [92,97,98,99]. Therefore, a nanomaterial-based approach for enhancing angiogenesis and osteogenesis must accelerate the BTR and ease the formation of an efficient, reliable scaffold.

## 3. Nanomaterials for Developing Bone Scaffolds

The nanoscale structure facilitates the bone-like structure with a ton of promising features. Nanomaterials integrated into the synthetic scaffold function as mechanical fillers; they are also employed to carry drugs and growth factors that trigger osteogenesis and bone regeneration [100]. A high surface area of nanomaterials enhances cell adhesion and communication, creating an optimal environment for nutrient and protein exchange. This structure offers adjustable strength and stiffness, which are essential for developing mechanical strength in synthetic bone applications (Table 1). Furthermore, the flexibility and toughness of nanomaterials can enhance the viscoelastic properties. The electric conductivity fosters osteogenesis by accelerating cellular signaling. Bioactive nanomaterials strengthen the microenvironment of stem cells, thereby promoting their differentiation. This process enhances osteoconductivity and osteoinductivity, which are crucial for bone formation through supporting cell attachment and proliferation. Cellular interaction is crucial for tissue regeneration and some nanomaterials offer a favorable surface for adhesion and migration. Controlling growth factor delivery ensures nutrients and metabolites that are required for the bone cell cluster reformation and prevents infection. In some instances, nanomaterials such as nanoparticles can be functionalized to carry the metabolites and macromolecules to the specific site of tissue. Some nanomaterials respond to external and internal stimuli to release the drugs and biomolecules for tissue growth. Post-surgical infection is another notorious challenge in regeneration systems, but nanomaterials possessing antibacterial and anti-inflammatory properties can enhance antibacterial activity and reduce inflammation following surgery. Lastly, a standard material scaffold should degrade in proportion to new bone formation, and the properties of some nanomaterials allow for controlled degradation through ion release, which further aids in promoting osteogenesis. Together, these merits earn the focus on nanomaterials to enhance the control over the regeneration of bone tissue. This section is designed to discuss the primary stem cells, along with the core polymeric nanomaterials and minerals used in BTR.

### 3.1. Inorganic Biominerals and Composites

Bone formation begins with the deposition of different collagen cells and is followed by the attachment of minerals to the skeleton for enhancing mechanical support and toughness [101]. Owing to their excellent resemblance to the natural bone components, the nanostructured inorganic minerals are considered potential candidates for synthetic bone grafting. In addition, the porousness of inorganic biomineral-based nanomaterials amplifies osteoconductivity and regeneration owing to their porous features. These minerals can be functionalized to load the drugs and release them sustainably. The size of the particle, enhanced tunability, surface area, porous surface and unique physical and chemical properties are important metrics in constructing a suitable candidate for the bone scaffolding [102]. Moreover, Table 1 presents an essential physical property of NMs for the BTE. Throughout this section, we will criticize the potentials of the inorganic biominerals and their composite.

#### 3.1.1. Calcium Phosphate Derivatives

Calcium phosphate-derived nanomaterials (CaP-NMs), including hydroxyapatite (HAp-NPs), tricalcium phosphate (TCP-NPs), and biphasic calcium (a combination of HAp-NPs and TCP-NPs), share a chemical similarity to the ECM of the bone. These components are biocompatible, nontoxic, bioactive, and osteoconductive in nature and are commonly used in BTE. Notably, Ca_3_(PO_4_)_2_-based cement is the first FDA-approved injectable biological cement that has been used in BTE [103]. Compared to the other NMs, CaP-NMs are favorably analogous to the bone minerals and they can be used both as intact and functionalized with the drug molecules to promote osteogenesis [104]. In addition, CaP-NMs can absorb a high ratio of biomolecules, which has led to their extensive application in BTE. They have been mostly taken up by the cells through endocytosis and analogous mechanisms. The following section will provide a comprehensive overview of CaP-NMs in the field of BTR.

**Figure 3:**
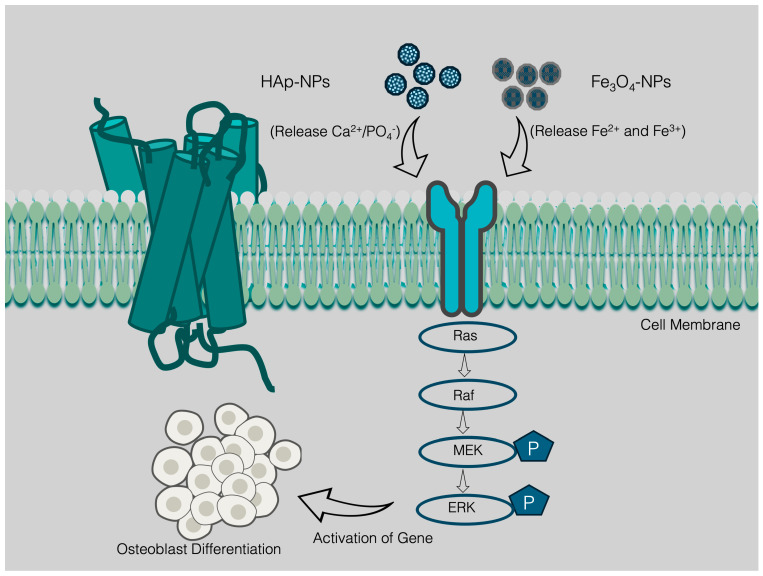
The activation of the MAPK/ERK pathway occurs in response to Ca^2+^ influx and mechanical stress, which is notably augmented by the presence of hydroxyapatite and Fe_3_O_4_ nanoparticles.

HAp-NPs (Ca_10_ (PO_4_)_6_ (OH)_2_) is one of the extensively investigated and well-recognized components in BTR technology. HAp-NPs drew attention since they are the largest inorganic constituent in mammal bones [105]. Nanostructured HAp is usually synthesized by exploiting wet chemical precipitation, hydrothermal, and sol-gel synthesis [106,107,108]. Compared to microstructural HA, HAp-NPs showed enhanced bioactivity, protein absorption, cell adhesion, biodegradability, non-toxicity, and resemblances with the ECM [109,110]. In bone regeneration, HAp-NPs reduce microleakage and reduce hard tissue denaturation due to their enhanced crystallinity [111]. In addition, HAp-NPs can be comprehensively used for specific targeting since they are bioactive, biocompatible, and have adjustable biodegradable characters [112]. Though having excellent potential, the osteoconductive properties of HAp-NPs are insufficient, but to circumvent the drawbacks, the NPs can be functionalized with several bioactive polymers (e.g., collagen, chitosan, cellulose, silk fibroin, alginate, gelatin) and GFs (e.g., GFs: BMP-2; VEGF; FGF-2, FGF-18) that will increase the osteoconductive properties [113,114]. A plentiful hydroxyl groups on HA eases the surface modification process and the modified NPs gain bonding with the synthetic and natural polymers through silanization and covalent bond formation [115]. The fabrication of an oriented polymeric scaffold has drawn attention because an increased number of these NPs can speed up the nucleation, which further helps create a biomimetic apatite [116]. The biocompatibility of HAp-NPs with natural and synthetic polymer modification enables higher mechanical stability and decreased immunogenicity and it overcomes the complex restrictions in bone scaffolding. Again, the nanostructured HA exerts anti-tumor potential and lessens cell apoptosis, which helps to trigger osteoblast proliferation in bone growth [110]. HAp-NPs induce the activation of the ERK1/2 MAPK, PI3K/AKT, as well as both canonical and noncanonical Wnt/β-catenin signaling pathways, thereby affecting osteoblast differentiation [92,117,118]. Figure 3 elucidates the ERK1/2 MAPK signaling pathway stimulated by HAp-NPs in the context of osteoblast differentiation.

Another calcium phosphate derivative is Ca3(PO4)2-NPs (TCP-NPs), which is broadly used in bone tissue regeneration and is predominantly remarkable for mimicking the natural bone phase and supporting new bone regeneration. TCP exists in α-TCP and β-TCP crystalline forms, where α-TCP is a crystalline with a monoclinic space group and β-TCP is rhombohedral [105]. The β-TCP form is frequently used in BTE owing to having slow degradation and enhanced stability [119]. β-TCP has a Ca^2+^ and PO_4_^2−^ ratio similar to that of natural bone. They release Ca^2+^ and PO_4_^2−^ for mineralization and bone matrix deposition, which are essential factors that contribute to osteoinductivity. The PLGA and β-TCP combination could be used as a filler and drug-substance carrier, enhancing osteoconductivity as well as 3D architecture for cell infiltration [120]. However, in BTR, TCP-NPs mimic the natural bone phase, while NPs are incorporated into the scaffold and coating mixture to interact with the surrounding tissue. It also improves osteoconductivity by stimulating mesenchymal stem cells (MSCs) to turn into osteoblasts and create a surface for attachment, proliferation, and differentiation [121]. TCP-NPs have enhanced cellular uptake ability and amalgamate finely to bone tissue [122]. Their enhanced porosity and interconnectivity feature provides endothelial cell migration and nutrient transpiration, ensuring vacuolization. Some studies reveal their cytotoxic explicitly and their aspect ratio is understudied in the BTE, but some investigations revealed that the flaws could be reinforced by integrating with the multi-walled carbon nanotube (MWCNT) and poly(lactide-co-glycolide) (PLGA) [123]. Upon amalgamation with the composite scaffold, TCP-NPs enhanced its toughness and mechanical stability. In addition, TCP-NPs can be functionalized with the GFs and supportive biomolecules for reinforcing angiogenesis and preventing infection [124]. Finally, in bone remodeling, their biodegradability feature makes them unique since it matches new bone formation, ensuring temporary support to the scaffold. At the same time, they are gradually replaced with the newly formed bone tissue to ensure that it does not hinder bone tissue formation [105].

Since TCP is proven to be the source that can accommodate great cell growth and HAP is reported to mimic the natural bone structure, the use of the biphasic concept has been developed to emphasize healing and BTR. This biphasic composition of HAp-NPs and TCP-NPs is known as biphasic calcium phosphate nanoparticles (BCP-NPs). Particularly, β-TCP is more used over α-TCP in HAp and TCP biphasic composition because BCP-NPs is reported to impart better bone growth, bioactivity, biodegradability, osteoconductivity, osteoinductivity, and compatibility compared to the CaP-NPs [125]. Combining NPs of these materials can increase bone healing better and faster. Some studies revealed that integration of BCP-NPs in hydrogel improved their biocompatibility and degradability, and that biomolecule-loaded BCP-NPs enhanced bone repair [126]. In addition to these minerals, calcium silicate, magnesium phosphate, and magnesium silicate have similar properties and contributing ability in bone scaffolding [11]. Conversely, the so-called low fracture toughness, brittleness, wear resistance, and low osteoinductive ability of HAP and TCP could be overcome with strategic compliance with their nanostructure and composite mixture with other biocompatible nanomaterials.

HAp-NPs facilitate signaling through the activation of receptors mediated by Ca^2+^, whereas Fe_3_O_4_ nanoparticles induce mechano-transduction. The observed effects culminate in the activation of ERK, which subsequently promotes cellular proliferation and initiates early osteogenic differentiation, thereby positioning them as significant assets in the field of bone tissue engineering. In the cell membrane, the influx of Ca^2+^ (HAp-NPs) and mechanical stress (Fe_3_O_4_) lead to the activation of integrins, calcium-sensing receptors (CaSR), and growth factor receptors, resulting in the activation of Ras. The cytoplasmic environment exhibits an activation of the phosphorylation cascade that includes Ras, Raf, MEK, and ERK. However, the mechanism coincides with a rise of Ca^2+^-dependent signaling, owing to nHA and an augmentation of mechano-transduction mediated by Fe_3_O_4_. In the nucleus, ERK initiates the activation of c-Fos and c-Jun, resulting in the assembly of the AP-1 complex. This process promotes the transcription of genes linked to cell proliferation and osteogenesis, consequently improving cell proliferation.

#### 3.1.2. Oxide-Based Nanoparticles

Oxide NPs have drawn attention since they aid in mimicking the inorganic component phase, producing a biomimetic environment and assist cell adhesion, growth, proliferation and differentiation. Primarily, they work as osteoconductive sources in the BTR. They contain rough surfaces that regenerate ECM and trigger the adsorption of cell adhesive proteins. The nanoscale structure facilitates the increased surface area to volume ratio, which qualifies their interaction with the cells and tissue, promotes osteogenesis, and contributes to BTR. In addition, they reinforce the mechanical strength of scaffolds and qualify them for the load-bearing function. Some oxide NPs demonstrate antibiotic potentials and prevent bacterial colonization at the defect site (Table 1). They also promote angiogenesis and enable the targeted and sustained release of the therapeutics controllably from the nanomaterials that are needed for the BTR. In this particular section, we will demonstrate the functional abilities of certain oxide NPs and discuss how they contribute to the BTR.

##### Graphene Oxide (GO), Reduced Graphene Oxide (rGO), and Composites

Graphene oxide and reduced graphene oxide are two-dimensional nanomaterials derived from graphene and are exclusively effective in the BTR for their enhanced tensile strength and flexibility. Usually, GO contains more oxygen-containing functional groups compared to rGO and the increased functional groups make GO a highly hydrophilic and well-dispersible agent. The disruption of the sp2 carbon network reduces its electrical conductivity and therefore GO yields semiconductor behavior [127]. Conversely, rGO is shaped from the GO and they undergo the reduction process that causes the elimination of oxygen-containing functional groups and restoration of the Sp2 carbon network [128,129]. Introducing GO composites into the bone scaffold induces osteoinductivity and osteoconductivity. They facilitated support for the osteogenic differentiation of human mesenchymal stem cells (hMSCs). hMSCs cultured in a 3D GO scaffold conserved cell viability, proliferation, and differentiation [130]. Their biocompatibility, large surface area and conductive microenvironment offer a valley for the culturing and maintaining of hMSCs [131]. The GO and its composite mixture (chitosan, polycaprolactone, hydroxyapatite) in the bone scaffold increase Young’s modulus, elasticity and tensile strength so that the scaffold gets extended mechanical integrity [132]. The addition of HAp-NPs into the GO and chitosan composite mixture achieved substantial endogenous bone regeneration, where GO and chitosan fostered the adhesion, growth, proliferation and differentiation of endogenous stem cells [129]. The osteoinductive ability of GO was revealed while adipose-derived MSCs were treated with the GO-NPs and the cell adhered, proliferated and maintained its properties [130]. Enhancing the mechanical strength is another must-marked property of GO. Collagen is one of the organic components of the bone matrix and is often used as a component in bone tissue regeneration since collagen is highly bioactive, biocompatible, and out of risk from immunogenicity, but it lacks mechanical strength. The inclusion of GO-NPs into the collagen strengthens the mechanical stability and pushes the creation of an ideal scaffold that can regenerate ECM [133,134].

Similarly, rGO contributes to BTR by amplifying the osteoconductivity in the scaffolds. They could be leveraged as their inclusion into the scaffold reinforces the mechanical properties, cell adhesion, proliferation and differentiation. The composite of rGO, HAp-NPs, and β-TCP boosts the tensile strength, which is one of the crucial features of osteoconductivity [135]. The rGO functions as a mechanical filler, bridging the cracks and thus amplifying their overall mechanical functioning [136]. Combined with other nanocomposites, rGO substantially improves stiffness, toughness, and load-bearing capacities and it upholds the adhesion and proliferation of osteoblasts in BTR. Endorsement of preosteoblast MC3T3-E1 cells alongside the HAp-NPs and rGO composite enables spontaneous osteoblast differentiation and contributes to new bone regeneration [137]. Prominently, the composite degradation in the scaffold is almost proportional to the new bone regeneration [129]. High surface area is another advantage coined by the rGO and this characteristic of rGO provides enhanced protein adsorption and cell microenvironment for growth and proliferation. Integration of rGO nanoparticles supports the differentiation of MSCs into the osteogenic lineage. In addition, a scaffold modified with the PLGA, HAp-NPs, and rGO coined a conductive microenvironment for the osteogenic stem cells and allowed delivery of bioactive molecules by mimicking ECM [138]. Therefore, despite having several spectacular advantages, the cytotoxicity of these NPs cannot be overlooked and concentration optimization of rGO must be conducted under superfluous precautions since rGO is more liable to interact with the membrane because rGO is comparatively hydrophobic and leads to the production of ROS following the oxidative stress to the cells and stops the replication. The cytotoxicity of GO is oriented by the apoptosis and necrosis of the cells since GO is implicit in endocytosis following interaction with the cytoskeletal component and nucleus of the cell [139]. For instance, a composite made from GO and chitosan reduced cellular toxicity and amplified the proliferation ability of osteoblasts [108]. Therefore, surface functionalization of GO and rGO with the biocompatible polymers and GFs potentially amplifies the osteogenic function of the scaffolds. In addition, long-term in vivo monitoring concerning the toxicity and safety related to graphene oxide derivatives must be embarked upon.

##### Magnesium Oxide (MgO) NMs and Composites

**Figure 4:**
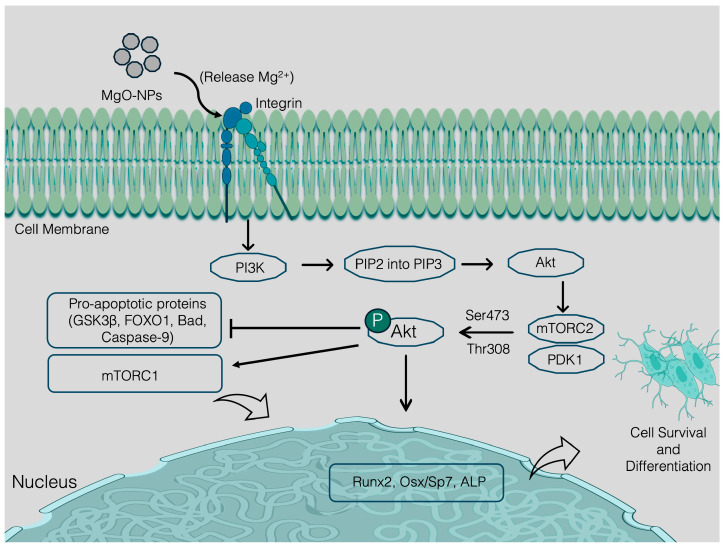
Magnesium oxide nanoparticles (MgO-NPs) improve osteoblast viability and stimulate bone formation through the activation of the phosphoinositide 3-kinase (PI3K)/Akt signaling pathway, leading to the inhibition of apoptosis and the enhancement of cellular proliferation.

Magnesium oxide is one of the primary components of bioglass, which features rapturous thermal and mechanical stability. It can dissolve in the biological fluid and after dissolving, they release Mg^2+^ ions in the biological microenvironment [140]. Mg^2+^ ions contribute to the bone mineralization and trigger osteoblast precursor gene for cell differentiation. Notable for their biocompatibility, biodegradability, and coordinating the osteoconductivity, magnesium oxide nanoparticles (MgO-NPs) garnered interest and they became a potential option for bone tissue regeneration. The favorable mechanical properties, such as tensile strength, have gained interest in BTR. The high surface area of MgO-NPs creates a microenvironment for interacting with the cells and proteins, thereby promoting osteoconductivity. Incorporating MgO-NPs into the substrate of the scaffolds magnifies the levels of alkaline phosphatase and bone mineral density. The increased level of alkaline phosphatase coins the activation of osteoblasts, following extended bone volume fraction. As a result of enhanced bone mineralization, bones become stronger and denser [141]. Mechanically, upon MgO-NPs dissolving into the scaffold, they release Mg^2+^ ions, which play a role in the Ca^2+^ metabolism mineralization of bone and contribute to osteogenesis [142,143]. Mg^2+^ solely possesses chondrocyte apoptosis through mitigating the PI3K/AKT pathway and inhibits the formation and function of osteoclasts through suppressing the AKT phosphorylation. Reversely, AKT phosphorylation activates the mTORC1 and suppresses pro-apoptotic proteins (GSK3β, FOXO1, Bad, Caspase-9). Activation of mTORC1 and suppression of pro-apoptotic proteins stabilizes osteoblast sustainability [144,145] (Figure 4). Osteoclasts and chondrocytes are accountable for bone resorption and cartilage formation [146]. Thus, the controlled release of Mg^2+^ is essential for controlling the homeostasis of osteoclasts and chondrocytes. MgO-NPs and their composites can safeguard a sustainable delivery of Mg^2+^ and GFs from the matrix and regulate the growth of these cell types for promoting osteoconductivity. Additionally, MgO-NPs have intrinsic antibacterial properties that lead to the inhibition of bacterial biofilm development through interfering with the metabolic process of the bacterial cell and disrupting their cell membrane. Their unique surface restricts the adherence and development of the bacterial biofilm, possibly possessing ROS that prevents the maturation of biofilm [142,147,148].

Conversely, due to the high dissolution rate of Mg^2+^, MgO-NPs tend to lose the Mg^2+^ ion while they are exposed to the biological solution for a long time. This orients a lack of mechanical strength of this NM. To prevent the premature dissolution rate, integrating MgO-NPs with the other nanocomposites synergistically excels the scaffold strength and mitigates the rapid premature dissolution rate [140]. For instance, surface coating on MgO-NPs with polymers such as graphene nanotubes (GNTs) and carbon nanotubes (CNTs) delays the degradation almost 2-fold and enhances mechanical stability [149]. The small diameter and extended mechanical hardness of GNT and CNT posed the protection against corrosion and bacterial and viral biofilm formation on the surface of MgO-NPs as well as other nanomaterials [150]. Again, immersion of the nanocomposite into the simulated body fluid led to interaction with the OH^−^ ion, developed magnesium hydroxide (Mg(OH)_2_) and contributed to the suppression of corrosion on the matrix. Mg(OH)_2_ develops a layer on the surface of the nanocomposite and simulates body fluid and substrate. The dense oxide layer prevents the inclusion of corrosive ions into the magnesium matrix [151]. Uniform dispersibility of MgO-NPs within the composite matrix contributes to fixing crack formation and ensuring structural integrity and longevity. This layer supplies the mechanical support needed for osteogenesis [152]. In addition, HAp-NPs/MgO composites show protracted microhardness, though their compressive strength is less compared to the bone matrix. Synergistically, MgO-NPs can reduce the limitation of HAp-NPs since the higher chemical stability of MgO-NPs prevents the aggregation, poor bonding strength, and melting point of HAp-NPs [153].

In the cell membrane, magnesium ions (Mg^2+^) bind to integrin receptors, leading to the activation of phosphoinositide 3-kinase (PI3K). The enzyme PI3K catalyzes the conversion of PIP_2_ to PIP_3_, thereby generating a docking site for Akt. In cytoplasm, Akt undergoes phosphorylation through the action of PDK1/mTORC2. Phospho-Akt exerts an inhibitory effect on pro-apoptotic proteins, specifically Bad and caspase-9, thereby averting cellular apoptosis. In the nucleus, Akt translocates to the nucleus, where it phosphorylates FOXO1, thereby inhibiting its pro-apoptotic activity. Ultimately, the systematic signaling pathways develop osteoblasts and the deposition of bone matrix.

##### Zinc Oxide (ZnO) NMs and Composites

Antimicrobial and osteoconductive ability garnered exclusive interest in the use of ZnO-NPs for bone tissue regeneration applications. The varying size of ZnO-NPs has been reported to form porous, bioactive, biocompatible, and biodegradable bone scaffolds. Additionally, ZnO-NPs foster collagen formation since Zn^2+^ facilitates the collagen cross-linking enzymatic catalysis and osteocalcin formation, which is the most crucial component of the ECM [154,155].

**Figure 5:**
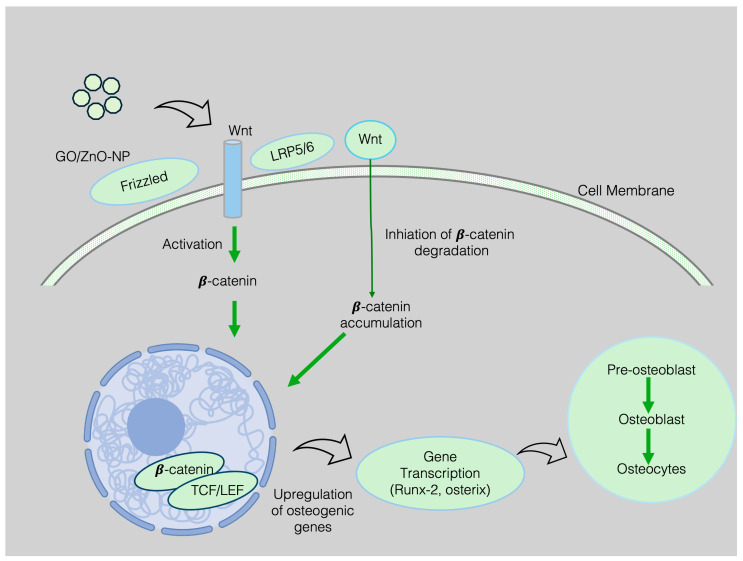
Graphene Oxide (GO) or Zinc Oxide Nanoparticles-mediated Wnt/β-Catenin Signaling Pathway. The activation of the pathway occurs upon the binding of Wnt ligands to Frizzled receptors and LRP5/6 co-receptors located on the cell membrane.

**Figure 6:**
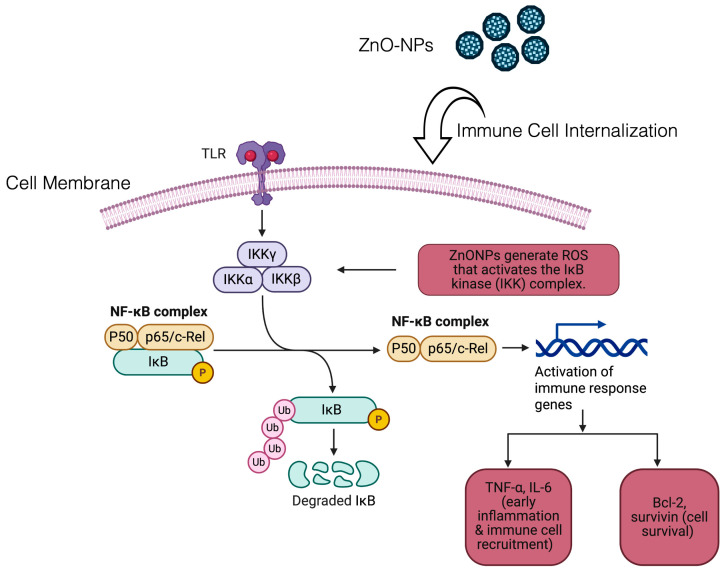
Zinc oxide nanoparticles induce reactive oxygen species (ROS) in cell cytoplasm. ROS initiates the activation of IκB kinase (IKK). IKK catalyzes the phosphorylation of IκB, thereby designating it for proteolytic degradation.

Primarily, the NPs trigger the osteoblast to develop the collagen and ZnO-NPs release the Zn^2+^ ion that contributes to the maturation and stabilization of the osteoblast through activating TGF-β/Smad and Wnt/β-catenin pathways. Briefly, upon the docking of TGF-β on cell surface receptor, they lead to phosphorylation that triggers the stimulation of Smad protein. Upon the activation, Smad proteins travel into the nucleus and recruit MSCs to the damaged bone site. Later, they differentiate and transform into the osteoblast. On the other hand, Wnt/β-catenin pathways are a cornerstone for deceiving cell fate. Wnt protein’s interaction with the Frizzled receptor mitigates the destruction of β-catenin. When β-catenin accumulates and enters the cell nucleus, it activates essential genes for osteoblast growth and maturation [156,157,158] (Figure 5). Besides that, NPs also enhance alkaline phosphatase (AP) activity, resulting in osteoblast mineralization and differentiation. The controlled release of Zn^2+^ stimulates osteoclasts and overturns osteoclast differentiation and mineralization through antagonizing NF-kB pathways (Figure 6) [159]. Also, these NMs are reported to trigger the PI3K/Akt and MAPK pathways that are acknowledged to alkaline phosphatase formation in the osteoblast regeneration process [155]. Indirectly, ZnO-NPs trigger the Ca^2+^ and PO_4_^3−^ ions, resulting in nucleation and mineralization of HAP formation for the osteogenesis [160,161]. In addition, the presence of NPs in the culture media can induce a microenvironment that is conducive to mineral deposition.

Moreover, ZnO-NPs possess excellent antimicrobial properties since they come into contact with bacteria, release the Zn^2+^ ion, and elicit oxidative stress or ROS in the bacteria [162]. The negative surface charge of bacteria is attracted by the positively charged ZnO-NPs in the aqueous environment and the electrostatic attraction leads to the accumulation of ZnO-NPs on the microbe surface. The deposition brings the zeta potential change of the microbial membrane, which destroys the potassium channel, following the lipid peroxidation, permeability, and cell blast [163]. While the cell membrane ruptures, the internalization of the Zn^2+^ ion travels inside and forms a complex with the thiol group. This complex interferes with the thiol-group-oriented enzymatic reaction, following the weakening of glycolysis and causing bacterial cell death [164].

In other folds, the inclusion of ZnO-NPs into the polymer-embedded scaffolds increases biodegradability and bioactivity. For instance, ZnO-NPs modified with polycaprolactone gelatine (PCL-G) creates a physical barrier that protects epithelial tissue by allowing periodontal cell and bone regeneration. At this point, ZnO-NPs augment the osteoblast marker and deposition of calcium in MSCs. PCL-G improves the microenvironment and stimulates osteogenic cell differentiation [125]. ZnO-NPs-cellulose nanofiber (CNFs) composite enhances the biocompatibility of the ZnO-NP and proposes a considerable composite in the BTR [155]. Overall, the composites favor the BTR, providing antibacterial strength, controlled degradation, and osteogenic stimulation. Having impactful qualities, concentration, size, and surface functionalization are critical parameters that need to be optimized to reduce adverse events.

Following Wnt activation, the destruction complex, which includes GSK-3β, Axin, and APC, is inhibited. This inhibition prevents the phosphorylation and subsequent degradation of β-catenin. Particularly, ZnO nanoparticles have demonstrated the capability to inhibit GSK-3β activity, which may occur via ROS-mediated signaling pathways or through direct interactions, resulting in the accumulation of β-catenin within the cytoplasm. The β-catenin/TCF/LEF complex within the nucleus drives the expression of osteogenic genes, including RUNX2 and Osterix.

Upon the degradation of IκB, NF-κB (p50/p65) is liberated within the nucleus. NF-κB undergoes translocation to the nucleus and interacts with DNA to modulate gene expression for producing pro-inflammatory factors (TNF-α, IL-6) or anti-apoptotic proteins (Bcl-2). These signaling pathways result in dual consequences. Inflammation is induced by NF-κB through the production of TNF-α and IL-6, while cell survival is promoted by the upregulation of Bcl-2 by NF-κB.

##### Titanium Oxides (TiO_2_) NMs and Composites

Titanium gained extensive attention owing to its corrosion resistance, mechanical stability, and biocompatibility. Their derivatives, such as titanium oxide nanoparticles, nanotubes and nanowires (TiO_2_-NPs, TiO_2_-NTs, TiO_2_-NWs), show high mechanical stability that can enhance long-term durability in the reformed bone in the BTR [33,62,165]. Synthesizing and fabricating titanium oxide (TiO_2_) nanomaterials follow electrospinning, gas-phase reaction, hydrothermal, anodization, laser ablation, and metal and gas-phase-assisted etching methods [166]. In BTR, TiO_2_-NTs is reported to foster the cell differentiation by activating BMP/Smad and integrin-mediated pathways. In addition, NTs enhance the expression of alkaline phosphatase and osteocalcin that triggers bone matrix formation [167]. The porous NTs facilitate nutrient transport and debris removal, fostering the new blood vessel that leads to angiogenesis. They also endorse osteoconductivity since they support bone-generating cells for attachment, growth, proliferation and differentiation, as well as osteointegration [61]. The conjugation of osteoconductive agents in the NTs surface boosts the osteogenic differentiation and the synergy leads to enhanced BTR [68]. However, the amalgamation of osteogenic cells with the TiO_2_ nanotube stimulates cell response, facilitates the delivery of biologics, and also provides excellent integration with the bone tissue, making them one of the unique contributors in the BTR [167,168,169].

Conversely, TiO_2_-NPs are bioinert materials with increased biocompatibility and permeability. Though TiO_2_-NTs have supportive osteoconductive landmarks, TiO_2_-NPs cause wear debris, weaken osteoblast function, promote osteoclast recruitment, and reduce bone integration, which further causes osteolysis and prosthetic friction, as well as implant relaxation [170,171]. Their photocatalytic properties were also brought to attention since high-concentration NPs induce oxidative stress. Under prolonged UV exposure, they generate excessive reactive oxygen species (ROS) and are proven to damage osteoblasts and proliferation, as well as cause apoptosis that reduces bone regeneration [171]. In addition, an increased concentration of NPs stimulates macrophages, and relative pro-inflammatory cytokines mediate inflammatory response. Chronic inflammation hinders the activity of osteoblasts, and its ultimate consequences delay bone regeneration. They also mitigate the emission of exosomes from the osteoblasts and reduce osteogenic differentiation [172]. Therefore, the flaws can be reduced or omitted through the fabrication of NPs with biocompatible polymers to enable their use in the BTR.

TiO_2_-NWs are another derivative of this group, which features exclusive mechanical, optical, and electric properties [173]. Their promising features make them a potential player in BTR. The NW’s high tensile strength and elasticity reinforced the scaffold structure since they can disperse the mechanical load throughout the wire, resulting in scaffold stress. Additionally, their flexibility grounds them to withstand mechanical deformation without breaking [174]. The high surface area aspect ratio directs the cell growth, bioactivity and biocompatibility, and antimicrobial properties enable them to contribute to the BTR [175]. Since they have a high surface area, they facilitate the dispersed sites for the cell adhesive protein and osteoblast attachment. The nanowire controls cell growth in a certain direction, which is important for the alignment of bone tissue and scaffold.

Most importantly, the scaffold can be formed by the self-assembly of nanowires and can grow in an upward–downward co-growth route, which is an easy and cost-effective source of TiO_2_-NWs [33]. Post-surgical events can contribute to reducing inflammation and infection since they can be functionalized with the GFs, genes, and other therapeutic agents on a large scale as they contain a large surface area [176]. Therefore, more rigorous studies and clinical trials are needed to optimize the use of TiO_2_ derivatives in BTR and implant strategies.

##### Silicon Dioxide (SiO_2_) NMs and Composites

Silicon dioxide nanoparticles (SiO_2_-NPs) have shown comparatively poor decomposition, reduced biocompatibility, and increased cytotoxicity. Thereafter, mesoporous silica NPs, composed of primarily SiO_2_ NPs, have been utilized with the polymers for bone tissue regeneration since meso/nanoporous nano-scaffolds offer improved restoration and functional recovery of damaged bone tissue [95]. Mesoporous silica provides an ambient microenvironment for cell growth, adhesion, and proliferation [177]. In addition, their proven biodistribution, assembly, and excretion ability place them as potential candidates for BTR [55]. The scaffold functionalized with the bone morphogenic proteins (BMPs) and other ligands exerts osteo-conduction as well as provides an environment for osteoprogenitor cell proliferations [177,178]. Integration of silica into the HAp lattice accelerates the osteoblast cell activity while Si^4+^ is released and the Si^4+^ ion triggers HAp nucleation and contributes to bone mineralization [179]. Composites of SiO_2_ have been used for multifunctional biomedical applications, including dental filling, bioimaging, and drug delivery [180]. Composites of soluble silica NPs and chitosan polymer work as a bone substitute. The composite led to osteointegration between the tissue and bio-composite interface [181]. SiO_2_ and GO nanocomposites in the poly-L-lactic acid (PLLA) bone scaffold have shown enhanced mechanical stability and biocompatibility in the scaffold [182]. In addition, functionalized SiO_2_-NPs with the recombinant human BMP-2 and integration into the CaPNPs scaffolds uprise osteogenesis and vascularization [183]. Again, gel beads made with collagen, glass nanocarriers, and mesoporous SiO_2_-NPs showed enhanced MSCs differentiation. SiO_2_-NPs were leveraged to load large quantities of fibroblast growth factor-18 (FGF18), resulting in the controlled secretion of FGF-18 from the hydrogel matrix following enhanced osteogenesis [184]. FGF-18 stimulates the ALP enzyme release that upregulates the gene expression for stimulating osteocalcin, osteopontin, and sialoprotein development [95]. In addition, a hybrid scaffold formed with PCL and SiO_2_-NPs enhanced the surface wettability gene expression for the osteogenic cell formation and mechanical strength, where SiO_2_-NPs functioned as a bio-interface and provided a cell-mitigating matrix. Mechanically, the nanohybrid extensively stimulated ALP, OPN, OCN, and col-1 genes for the resulting matrix, which promoted mineral apatite formation, MSCs growth, and time-dependent differentiation. This nanohybrid also contributed as a theragnostic agent since they were capable of loading high quantities of cytochrome C, gentamycin sulfate, fluorescein isothiocyanate, and doxorubicin on their surface and, amazingly, showed sustained release ability [185]. Therefore, the ease of surface functionalization and loading ability of SiO_2_-NPs enables them to be the osteoconductive and osteoinductive agents in the BTR. These NPs can be modified and cross-linked with the other synthetic and biogenic polymers, thus enhancing their ability to carry large quantities of drug molecules and have a controllable release, including GFs and antibiotics that effectively contribute to BTR.

##### Magnetic Iron Oxides (IO) NMs and Composites

Magnetite (Fe_3_O_4_) and maghemite (γ-Fe_2_O_3_) are two primary forms of magnetic iron oxide. Their NPs possess intriguing superparamagnetic properties, which imparted their title to superparamagnetic iron oxide nanoparticles (SPIONs). SPIONs could be efficiently functionalized with the bioactive molecule for targeted delivery and monitoring. Notably, SPIONs are FDA-approved standard contrast agents [186,187]. The super-magnetism, biocompatibility, and catalytic ability of these NPs qualify them for multifunctional application in biomedical and BTR technology. Exclusively, they respond to the magnetic fields and act as a source of drug delivery agents [108,188]. IONPs modulate the performance and functional behavior of bone-regenerating stem cells. They have been leveraging MSCs for labeling and tracking the cell in the BTR [189].

Current BTE uses the inherent magnetism of SPIONs in the cellular microenvironment. Their superparamagnetic property facilitates direction in osteoinductive, osteoconductive, osteogenesis, and angiogenesis in scaffold formation. IONPs or other magnetic nanoparticles can be integrated into scaffolds by leveraging electrospinning, freeze-drying and covalent linkage. They increase hydrophilicity and surface roughness for harnessing cell and protein adsorption [190]. However, the excessive concentration may suppress the mechanical properties of the scaffold, but optimized concentration surpluses the stiffness. These NPs accelerate cell proliferation by creating a microenvironment and providing a high surface area that harnesses the ion channel activation, Wnt (Figure 5), and BMP2/Smad/Runx-2 signaling pathways. The activation of mechanosensitive ion channels and signaling pathways accelerates osteogenic marker formation as well as osteogenesis [191,192]. The results of the in vivo experiments reveal that using SPION-loaded scaffolds demonstrates greater mineralization and integration with the host tissue for bone formation [193,194,195].

Furthermore, they foster vascularization through allowing nutrient transport and removing debris. Also, the magnetic microenvironment created by SPIONs can trigger the release of vascular endothelial growth factors (VEGFs) [187] (Figure 3). They also trigger osteoblasts to secrete the molecules necessary for endothelial cell function [189,196]. It enables the remote delivery of biomechanical stimuli and controls cell retention in a certain place where they are needed to speed up the healing process [187]. Stimulation of SPION-loaded osteoblasts with the external magnetic field causes stretching and bending in the scaffolds and this force incites macrophages to consume an increased amount of oxygen, resulting in the release of angiogenic growth factors and the formation of new blood vessels [196]. SPOINs are used as a negative contrast agent, enabling imaging of the MSCs survival in the cartilage graft, eliciting the prospective function for osteo-angiogenesis [197]. However, applying to an external field is not cost-friendly and needs expertise to amplify the process. Internalizing the NPs raises toxicity concerns and high concentration leads to cell toxicity since they form free ions within the cells that cause ROS and produce oxidative stress to the cell [198] while we believe polymer cross-linking, surface modifications and optimized SPION concentration in the scaffold can unscramble the issue.

#### 3.1.3. Polymers and Polymeric Nanostructure

Polymer-based materials are cornerstones in bone tissue engineering and the number of materials used as adjuncts to BTR has risen rapidly over the years. BTR often uses polymeric scaffolds for reliable physical support, but before their amplification, optimization is a crucial concern for obtaining the desired constraint. Key limitations such as biodegradability, cytotoxicity, and processability persist in metal and ceramic materials and urge the integration of polymeric materials into BTR. Polymeric nanomaterials escalate the mechanical strength of the scaffolds, and their large surface area has the capability of loading a high quantity of bioactive molecules directly into the scaffold for enhancing functionality and accelerating BTR. They are tunable in appropriate size, shape, and architecture to resemble certain bone defects [199]. A significant number of functionalities can be conferred on synthetic and natural polymers to adapt mechanical stability, porosity, environmental sensitivity, and biodegradation rate [200,201]. Typically, polymers breed protection from immune attacks and their enhanced conductivity and increased tunable surface chemistry provide compatible integration with the neighboring tissue. However, two major types, including synthetic and natural polymers, are widely used in the bone tissue regeneration [202]. Natural polymers are capable of mimicking ECM, are favorably biocompatible, highly biodegradable, and can be utterly replaced by nascent bone, but they lack mechanical strength and thus degrade promptly [203,204]. Their restorability is grounded on their hydrolytic and enzymatic degradation [103]. Synthetic polymers are also relatively hydrophobic, but hydrophilic synthetic polymers are also available [205]. Importantly, their surface could be functionalized to deliver essential bioactive molecules, which are easy to functionalize, tunable, and easily processable. On the contrary, they possess less cell adhesion, but surface modification could enhance cell adhesion and growth [206].

Furthermore, their topology can be modified for steady degradation that aligns with the growth of tissue, and customized porosity and mechanical properties make them suitable for BTR [202]. Their tunable shape, size, biocompatibility, and immunoprotective nature profuse the potential of synthetic polymer in the BTR field. Furthermore, the nanoscale structure of these polymers plays a substantial role in the BTR. The nano topology, including nanopores, nanoridges, and nanogrooves ranging from 1–1000 nm in dimension, can effectively guide osteoprogenitor cells for differentiation into bone cells towards the osteogenic linage [207,208]. The topology of NM features with the natural bone-like structure facilitate mechanical cues to the osteoprogenitors that they can sense and respond to by adhesion, distribute throughout the surface, and finally differentiate into bone cells [207,209,210]. In this section, we will demonstrate merely US-FDA-approved synthetic polymers as well as their nanostructure with their specific application in BTR.

##### Polylactic-Co-Glycolic Acid (PLGA) and Composites

Polylactic-co-glycolic acid (PLGA) is often used to develop therapeutics, including drugs and biomolecules, in bone regeneration. Its versatility and outstanding features acknowledge this synthetic polymer in developing nanocarriers to effectively encapsulate large quantities of drugs and biomolecules for delivery and release controllably in a specific site of bone defects [211]. The US Food and Drug Administration (US-FDA) and European Medicines Agency (EMA) approved PLGA for drug delivery purposes [212]. Since cytotoxicity is one of the key obstructions and is related to safety concerns, the PLGA-NPs were administered orally to mice and a study was conducted to elucidate their biodistribution and cytotoxicity. Results demonstrated that NPs gathered in the kidney, liver, and brain, but there were no signs of tissue necrosis or inflammation, making them safe for in vivo application [213]. Primarily, the polymer is exceptionally biocompatible since its lactic and glycolic acids are the only metabolites that can be simply excluded from the metabolic cycle since the body effectively deals with monomers and exerts minimal/no toxicity [214]. PLGA is generally synthesized with polycondensation and ring-opening co-polymerization [215,216,217]. During the synthesis, the ratio of lactic acid (LA) and glycolic acid (GA) can be simply adjustable; thus, the degradability of PLGA-NPs can be extended to a few months, which is consistent with the bone structure remodeling as well as bone regeneration [218]. For instance, using double-layer PLGA-integrated scaffolds attained synchronous healing of osteochondral tissue where two distinctive scaffolds were made with different pore sizes and merged to create an integrated scaffold. The outcome revealed comparably better osteogenesis compared to the usual bone scaffold [219]. The porosity of the scaffolds is another crucial concern; while mimicking a scaffold, a porosity range of 70–85% is considered excellent for creating osteoinduction in cancellous bone. A PLGA scaffold with directional holes could be created with 90% porosity and strong mechanical strength. The consistency and directional migration of stem cells throughout the PLGA scaffold is obtained, which has a favorable influence on BTR [220]. Again, the inclusion of platelet-rich plasma that contains a significant amount of GFs and proteins in the biolayer PLGA scaffold improved the growth of BM-MSCs following the reconstruction of osteochondral defects [221]. Similarly, seeding adipose tissue-derived MSCs (AD-MSCs) on the PLGA scaffold refurbished the skull bone since PLGA facilitates a better surface for the differentiation and proliferation of osteoblasts and chondrocytes in rat models [222]. In addition to the aforementioned benefits, PLGA combined with other polymers reinforces the osteoconductive properties in the scaffold. Adding VEGF to the gel of PLGA and methoxy-polyethylene glycol (mPEG) enhanced local vacuolization and bone regeneration [223]. Integration of TiO_2_ in the PLGA scaffold reinforces Young’s modulus to the PLGA, which could enable fine-tuning for the BTR application [224]. Again, PLGA/TCP/Mg, PLGA/HA, BG/collagen/PLG and PLGA/β-TCP composites substantially enhanced tensile integrity and biocompatibility, retaining mass degradation scaffold over time, which indicates the reinforcement of osteogenesis and osteogenesis [219,225,226,227]. Doxorubicin and isoflavones have been incorporated into the PLGA/TCP scaffold for recovering from post-bone tumor resection surgery, where the scaffold results in bone regeneration and tumor cell suppression [78,228]. However, having the aforementioned efficiency, hydrophobicity is limited in PLGA and using PLGA merely in a scaffold might impede efficient protein and cell adhesion as well as cell seeding.

On the contrary, PLGA-NPs are well recognized for showing several etiologic benefits, including tunable surface functionalization and controllable hydrophobicity. The difficulty can be resolved by covering the scaffold surface with the artificial ECM, usually composed of collagen type 1, fibronectin, vitronectin, hyaluronic acid sulfate, and chondroitin sulfate or fibrins [215,229]. In addition, PLGA-NPs are prone to aggregate and burst promptly upon exposure to the biological fluid. Overcoming this issue can gain more acceptance of PLGA-NPs for BTR and the ratio of LA and GA needs to be optimized to augment the biodegradability of the scaffold.

##### Poly Lactic Acid (PLA) and Composites

Poly Lactic Acid (PLA) is another US-FDA-approved aliphatic polymer that is repeatedly used in BTR since the polymer is biocompatible and has good processability. They could be designed in multiple ways, depending on the type and shape of the bone scaffold [230]. Importantly, PLA-NPs could be synthesized from renewable sources, including cornstarch and sugarcane and the polymer has three stereoisomers, such as poly L-lactide, poly D-lactide, and poly DL-lactide [231]. The crystallinity of the PLA has a significant impact on the scaffold’s mechanical stiffness, modulus, hardness, tensile strength, and melting temperature, as well as degradation. Crystallinity and thermal stability depend on the choice of stereoisomers, their distribution, molecular weight, and thermal processing [232,233]. In addition, molecular weight also has greater impacts on the solubility and the degradation period and low molecular weight is preferable in the scaffold preparation and tissue engineering since low molecular weight PLA facilitates an almost parallel degradation period with the nascent bone regeneration, whereas increased molecular weight may take longer absorption and degradation time following the orientation of infection and inflammation [230,234,235]. Remarkably, the elasticity of the PLA modulus is similar to that of human bone [233]. Intriguingly, PLA degrades following surface erosion, where the outer surface of the polymer is exposed to the water interface first and gradually wears away the internal surface. They also follow the bulk erosion mechanism of degradation, where the polymer degrades uniformly into carbon dioxide and water molecules by the hydrolysis of ester bonds present in PLA [236]. Modification or copolymerization of PLA improves the biological function and properties in BTR applications since copolymerization with selective materials extrapolates and refines the chemical composition, morphology and tropology and sometimes yields synergism [237]. For instance, a composite mixture of PLA and PGA decreases the crystallinity melting temperature [238].

3D-printed scaffold designed with HA/PLA composition enforced the cell adhesion and proliferation, therefore overcoming the low cell adhesion issue associated with the PLA. Hence, HA exerts electrostatic interaction with the cell receptors, and the composition creates a balanced scaffold for regenerating bone tissue [239,240]. In addition, PLA/β-TCP enhances ossification and adhesion [241]; PLA/Mg shows antibacterial properties and improves mechanical properties [242]; PLA/TiO_2_ leads ductility, including corrosion resistance, as well as shows effective antibacterial properties [243]; and PLA combined with natural polymers creates a surface for adding ligands for optimized cell adhesion and effectively promotes osteogenesis [244]. Recently, an antibacterial product has been developed using PLA fiber/poly(ε-caprolactone) loaded with vancomycin that was purposely prepared and showed efficient wound repair [245]. Though PLA provides immense application scope, PLA also bears limitations, including limited cell adhesion and osteoconductivity and is prone to acidic discharge in biological environments that might possess inflammatory reactions [230]. In addition, blending with other polymers may generate incompatibility since PLA’s aliphatic and hydrophobic nature leads to a decrease in the functional properties of the composite or co-polymers [246]. Additionally, fluorination or adding a hydrophilic copolymer on the PLA surface sustainably increases the cell adhesion at their surface, but gas-phase fluorination introduces C-F bonds into the backbone of PLA and shifts the surface hydrophilicity and polarity to facilitate better cell adhesion and distribution on the PLA surface [245].

##### Polyglycolic Acid (PGA) and Composites

PGA is another aliphatic polymer of glycolic acid, one of the most commonly incorporated and finest biodegradable polymers, which can be used for bone scaffolding. Notably, PGA is the only hydrophilic, biodegradable, and FDA-approved polymer [247]. The human body produces glycolic acid at the event of metabolism and the polymer degrades by hydrolysis of the ester bond and the resulting monomer either enters into the tricarboxylic acid cycle or is eliminated from the body through urinary excretion [248]. Compared to other biodegradable polymers, PGA is well suited in BTR due to its increased crystallinity with Young’s modulus of 7.0 GPa and its mechanical strength is similar to that of normal bones [249]. In addition, PGA is nontoxic to the cells and mends cell adhesion, growth, proliferation, migration, and differentiation for faster BTR [250]. Conversely, the rapid degradation limits the mechanical potential of PGA and due to its high glycolic acid content, the polymer may induce an inflammatory response. In addition, they do not have a favorable surface for adhesion and proliferation due to the lack of a specific seeding site and the release of glycolic acid upon degradation. However, adding copolymers and surface modification combats the problem [247]. Therefore, several studies claim the potential of PGA, certainly in BTR; for example, different ratios of PGA/β-TCP achieved effective, rapid, and stronger osteoconductivity in femoral bone defect and consequential natural bone in the integrated area [247]. Mastering PGA with silk fibroin (SF) fiber protein-based scaffold enforces cell adhesion and proliferation to its surface, resulting in osteogenic differentiation in both in vitro and in vivo settings. The material combination helps create vascularization that is essential for carrying oxygen and nutrients throughout the blood vessel [251]. Another study was conducted to evaluate osteoblast behavior leveraging arginine, aspartic acid, and glycine-functionalized PGA fiber and cell morphology, proliferation, and calcium phosphate deposition were measured. The study reveals a significant number of cell viability and an elevated quantity of Ca_3_(PO_4_)_2_ in the osteogenic inductor medium, which is a wholesome indication for the new bone matrix formation [252]. In addition, a multilayer scaffold consisting of HAp/γ-PGA and collagen/γ-PGA was made using two printheads of a 3D bioprinter. The resulting ceramic and polymer matrix scaffold showed effective biodegradation and the mechanical properties increased fivefold compared to the 2D (single-layer) membrane scaffold. The in vitro and in vivo results indicate the presence of PGA and multilayer scaffolds provide more possibilities for ostochondrogenesis [253]. Another study demonstrated scaffold formation by integrating interleukin-4 (IL-4) and TCP into the PGA-SF core-shell to obtain the prompt release of IL-4 and sustain the release of TCP. Purposely, IL-4 upregulates M2 macrophages for combating inflammation and, therefore, the PGA-SF/TCP/IL-4 scaffold synergistically creates a favorable microenvironment for providing osteogenesis and mineralization for achieving bone reconstruction [254]. In addition, nanostructure PGA provides a wide surface area and porosity for drug, GFs, and other biomolecule delivery. Similarly, nanostructures of PGA can be used to achieve tunable degradation rates that match nascent bone formation. The nano topology guides osteoprogenitors towards osteogenic lineage by facilitating mechanical cues for attaining cell differentiation and osteogenesis [207].

##### Poly Caprolactone (PCL) and Composites

The unique combination of bio-absorbability, biocompatibility, and fortified mechanical strength emerged in PCL for becoming a critical biomaterial in BTR applications. The FDA approved this synthetic polymer for multifunctional uses, especially in biomedical applications, which are well pronounced. This aliphatic and semicrystalline polymer extends the support for the cell adhesion osteogenesis and while integrated into the bone scaffolds, it degrades at a rate of bone regeneration process [255]. Upon exposure to the biological fluids, the polymer hydrolyzes into the monomer through breaking the ester linkage, though it takes around 2–3 years [256]. In addition, the glass transition point of PCL is approximately −60 °C and the melting temperature point is 60 °C, which enables its use in specific 3D printing and biomedical applications [257,258]. Low glass transition and melting points indicate their elastic behavior at room temperature, enabling the formation of many tunable structures. In addition, its cost-effectiveness, gradual degradation, rheological properties, viscoelasticity, non-cytotoxicity, incredible drug permeability, and ease of processability make it open for long-term clinical and biomedical application [256]. With upheld application and effectiveness, PCL bridges the mechanical or structural steadiness and bioactivity, therefore offering a versatile component for BTR application.

Many studies on PCL, its composite and nanostructure forms, have already been executed to mine their inherent possibilities in advancing BTR. Despite having a good number of possibilities in BTR, PCL is hydrophobic by nature, which sets borders, including less cellular attachment, growth and proliferation [259]. PCL is most commonly used in its modified form with specific features so that overcoming its inherent limits, and similar to other synthetic polymers, PCL can undergo surface modification and fusion with other hydrophilic polymers to improve their cell adhesion and proliferation capability [260]. In addition, the integration of nanoparticles into the PCL matrix can literally alter the inherent properties of net PCL and extend the biomedical application. Since pure PCL polymers are continuous, smooth, and uniform, the incorporation of HAp-NPs results in a rougher fiber surface, which is crucial for mimicking ECM. Compared to neat PCL, the nanocomposite showed extended elongation and surface area for cell seeding. Thicker fiber with reduced agglomerate fiber creates a favorable environment for cell adhesion and differentiation and can be produced from this nanocomposite [261,262]. The composite prepared with PCL/MgO and PCL/chitosan/MgO using electrospinning enhanced the tensile strength and induced a rougher surface for cell adhesion. Though the PCL/MgO composite enhanced Young’s modulus, adding chitosan to the pure PCL decreased the modulus from 21.6 to 6.8 MPa [263]. Another study showed that the inclusion of cellulose nanocrystals (CNCs) into the PCL matrix surpluses the hydrophilicity and overall wettability of the matrix. Adding CNCs also increased the tensile strength as well as Young’s modulus [264]. They also lionize the cell viability and enhance osteogenic differentiation, which indicates that the composite is biocompatible and non-cytotoxic for bone scaffolding [265]. Composites composed of PCL and silver nanoparticles (AgNPs) led to the size reduction of the PCL nanofiber and the reduced size is substantial for enhancing surface charge density. PCL does not exhibit any antimicrobial effects, though the composite fiber demonstrated significant antimicrobial aptitudes. The same study also demonstrated that only PCL and PCL/AgNP composite are non-toxic to the scaffold and 0.2 mM AgNPs in bone scaffolds achieved higher stem cell viability [266]. In addition, a PCL/gelatin scaffold with multi-walled carbon nanotubes (PCL/gelatin/MWCNTs) improved surface wettability and reduced the diameter of fiber size. The aligned distribution of MWCNTs contributed to the enrichment of tensile strength compared to the PCL/gelatin blend, which created the scaffold solely. Though the PCL/gelatin blend, as well as PCL/gelatin/MWCNTs composites, supported the cell adhesion, a scaffold with enhanced surface wettability and smaller diameter magnitude additional cell proliferation [267]. In addition, PCL/β-TCP, PCL/gelatin, and PCL/chitosan composites reveal accelerated cell adhesion, retention, and mechanical support to the bone scaffold [268,269,270]. Therefore, in bone tissue regeneration, PCL, PCL-based blends and copolymer composites offer more advantages compared to neat PCL.

## 4. Regulatory and Commercial Aspects of NMs-Based Scaffold

The regulatory affairs concerning nanomaterial-based scaffolds can be a long and expensive, and tedious process and must be addressed before it can be commercialized. Researchers are keen to follow the standards set by established regulatory authorities when it comes to commercializing and advancing biomedical research using nanomaterials. However, progress has been significantly slowed down due to the regulatory departments’ heavy workloads and lack of interdisciplinary communication and understanding. Alternatively, the marketplace has its own level of expectation regarding the fundamental use of these hybrid methodologies. Therefore, there are no finite US-FDA/EMA guidelines, GMP scalability, or large-scale in vivo testing comparative databases that set the precedence for standardization of NM-based BTR. FDA has published several guidance documents regarding the application of nanotechnology in FDA-regulated products and uses existing review processes and classification of products based on different risk classes to assess products on a case-by-case basis. Yet, there is not much specific guidance for NM-based BRT. The persistent lack of long-term preclinical and clinical translation for monitoring the decade-long toxicity, carcinogenicity, and excretion of NMs has remained a pivotal challenge. This has sparked concerns about the industry production and commercialization of NMs-based scaffolds for the BTR. In addition, given the lack of predictive computational models for NMs behavior in complex scaffold matrices, the unknown scalability of NMs hinders the overall research progress. In addition, the lack of standardized toxicity index of NMs on in vivo or in rigorous physiological conditions is impeding the success of clinical translation [271]. Hence, testing NMs for the BTR application would need to undergo a series of case-by-case reviews and the implementation of a standard safety protocol for accelerating the clinical translation. Global regulatory authorities must be more unified and transparent in order to speed up the licensing protocol for NM-based scaffold formulation. Additionally, a standard testing procedure for long-term safety and effectiveness must be established by the global regulatory authorities. Early involvement with the regulatory authority to classify raw material can streamline the approval process.

On the positive side, a growing number of commercialized nano-based bone scaffolds have been developed and brought to market in recent years with FDA-approval—for instance, the US-FDA approved NanoFUSE which consists of demineralized bone matrix (DBM) and 45S5 bioactive glass and has been in use to address bony voids. In exposure to the biological fluid, NanoFUSE releases Ca^2+^, Na^+^, Si^2+^, and PO_4_^3−^ ions which increase the pH and subsequently activates hydroxyapatite and cellular activity [272]. Likewise, Actifuse is an FDA-cleared nanomaterial-based scaffold that surpasses traditional HAp in terms of fusion; nonetheless, it was withdrawn due to market competition [273]. NanoBone Putty is another milestone of scaffold formulation success where HAp is embedded in a highly porous silica gel matrix, giving this implant enhanced osteoconductivity, early cell attachment, and controlled biodegradation [274]. Last not least, OsteoFlo-NanoPutty is a synthetic bone graft that leverages nano-surface technology and a quad phasic composition—comprising bioglass, α-TCP, β-TCP, and hydroxyapatite (HA) to promote cellular activity and osteointegration through a regulated and synergistic resorption process.

NanOss, Op-1 Putty, and OSTEOTRANS-MX were unsuccessful due to unwanted outcomes. Briefly, NanOss was intended to increase the surface area of the scaffold and replicate the bone mineral phase but this technology failed in preclinical trials due to a mismatch between the tissue growth and degradation kinetics [275]. Despite failure in preclinical trials due to the aforementioned mismatch between the tissue growth and degradation kinetics, the optimized NanOss 3D Plus incorporates the nanostructured super-fine HAp crystals designed to mimic the natural bone surface by design, and has achieved a high fusion rate of 97% [276]. Furthermore, Op-1 Putty was intended to deliver recombinant BMP-7 in a controlled manner. Essentially, collagen/carboxymethyl cellulose was used to establish the matrix of the scaffold which had an unfortunate outcome causing insufficient mechanical stability, uncontrolled release of recombinant BMP-7, and uncontrolled immune response. OSTEOTRANS-MX is a biphasic scaffold that was primarily formulated with inorganic and organic nanomaterials. Failure in the homogenous distribution, pore inconsistency, and uncontrolled degradation rate of NMs led to the failure of this product [277].

Therefore, successful preclinical and clinical translation, market acceptance, and a path to regenerative medicine market demand is tied to the commercial growth of NM-fabricated scaffolds. The global market for the regenerative medicine market is estimated to be worth $43.80 billion in the current year and is expected to expand to reach $212.80 billion by 2034. Tissue engineering has acquired 28.84% of the overall market in 2024, highlighting scaffolds’ significant and growing demand. According to Coherent Market Insights, the global scaffold technology market is predicted to develop at a compound annual growth rate (CAGR) of 12.7%, from an estimated $2.31 billion market this year to $5.34 billion by 2032. Scaffold-based methods are expected to share 42.9% of the overall tissue engineering market in 2025 (https://www.ihealthcareanalyst.com/global-regenerative-medicines-market/; accessed on 29 July 2025).

## 5. Unmet Challenges and Future Opportunities

**Figure 7:**
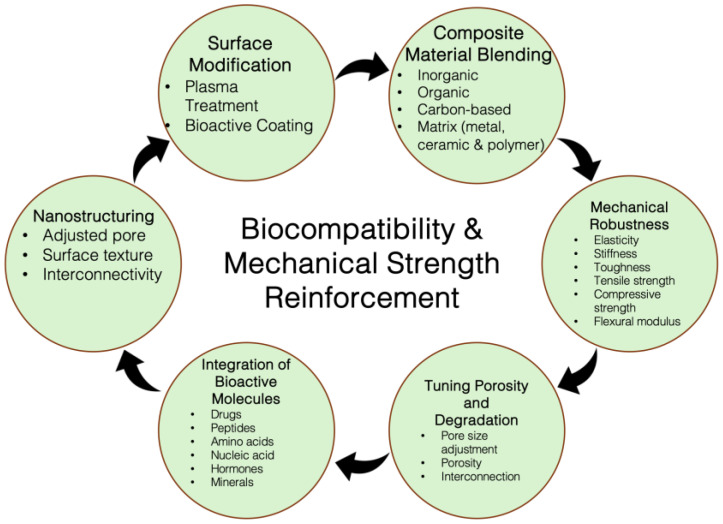
Future prospects for reinforcing biocompatibility and mechanical properties of NMs-based Scaffold. Here, plasma treatment aims to alter/introduce the functional group to the surface and bioactive coatings aim to mimic the ECM. A composite blend tends to blend biocompatible inorganic, organic, polymer, ceramic, or carbon based NMs for achieving combined beneficial properties. Mechanical robustness certifies a scaffold’s ability to withstands stresses and forces. Bioactive molecules reparents the cell proliferation and regeneration supportive drugs (e.g., anti-biotics; prostaglandins; statin; bisphosphonates, dexamethasone;), polypeptides (e.g., bone morphogenic proteins; platelet derived growth factor; vascular endothelial growth factor; fibroblast growth factors; transforming growth factors; insulin-like growth factors), amino acids (e.g., essential amino acids), hormones (growth hormones; calcitonin; parathyroid hormone; calcitriol), mineral (calcium; zinc; silicon; strontium; fluoride; magnesium), nucleic acid (miRNA; siRNA). Pore size, porosity, and interconnectivity among the pores are always important since a scaffold’s mechanical strength, cell migration, and vascularization depend on these parameters. Definite surface textures promote cell attachment, migration, and differentiation into the scaffold matrix. Proper use and experimental design will reinforce the production of a biocompatible and mechanically strong scaffold.

The science of nanomaterials in BTR stands at the intersection of biomaterials, molecular biology, immunology, and regenerative medicine. Along with the technological and biomaterial development, several pitfalls exist in the field. Despite the immense importance of NMs, this sector still has a number of notable research and knowledge gaps. Primarily, the aggregation of NMs remains a substantial barrier in nanocomposite development for the BTR, which is substantially mitigating their functional and mechanical performance. For instance, the tendency of GO or HAp-NMs aggregation has occurred through the van der Waals and hydrophobic interactions, resulting in the collapse of electrostatic repulsion among the particles, following the aggregation under physiological conditions. Across from that, NMs tend to form protein coronas in biological fluids where serum proteins develop clusters with the NMs that result in the agglomeration [278]. Thereupon, the agglomeration changes the NM’s actual size and shape, perhaps defeating the purpose of their use. In addition, agglomeration can cause the burst drug release instead of the intended controlled release. These consequences might bring detrimental defects to the scaffold. Consequently, burst drug release causes the deprivation of cellular infiltration and will detrimentally trigger the immune response that would cause the fibrous infiltration in lieu of tissue integration. Albeit to resolve these issues, concurrent studies have focused on NM surface modification strategies. PEGylation and zwitterionic coating have been successfully introduced in several studies, and they prominently minimized protein coronal and NMs aggregation [279]. Zwitterions form a hydration layer and form steric repulsion that prevents NM aggregation in solution. PEG is a long-chain hydrophilic polymer. The PEGylation on NMs crafts a conformational cloud around the NMs. This conformational cloud creates a steric hindrance and introduces a hydrophobic barrier among the NMs. In addition, PEGylation reduces the electrostatic and hydrophobic interactions among the NMs. In addition, the layer of PEG can produce a stealth effect that acts as a shield between the NMs and protein corona in a biological environment [280]. Besides these critical approaches, matrix-optimizing approaches such as hydrogel shear thinning and dispersion techniques such as pulsed electromagnetic fields can be applied for preventing the NMs clustering in scaffold applications. Furthermore, the complicated actions of NMs on immune cells are still not fully understood. The foreign body reactions, which are often initiated by the immune system attacking the bio/nanomaterials, are highly concerning. This scenario exacerbates over time while a foreign body reaction starts encapsulating the synthetic bio-scaffold. Although surface modification with the immunoprotective biomaterials and immunomodulatory peptides has been leveraged to cover the surface of nanoscaffolds, the cost-ineffectiveness hinders the equitable access. Additionally, non-degradable NMs often generate ROS amid deposition in vital organs, consequently orienting several tissue toxicities [281]. Therefore, Figure 7 provides an overview for reinforcing biocompatibility and mechanical properties of NMs-based Scaffold.

Along these lines, we have briefly covered the current and future aspect of NMs in BRT for astronauts regarding its use in space sciences because bone regeneration will become a major concern as the length of space mission times are extended, particularly in the near-future endeavor of Martian colonialization. Microgravity is the condition in which people or objects appear to be weightless. The effects of microgravity can be experienced when astronauts float in space aboard the International Space Station (ISS) and among manned orbiting spaceships. Astronauts within long-duration flight missions experience extended microgravity effects on the human body. These effects include bone and muscle loss, cardiovascular changes, and immune system alterations, to name a few. These effects are due to the absence of Earth’s gravitational force. The longer astronauts are suspended in microgravity, the more bone they lose, and the more challenging it becomes to mitigate these effects with current countermeasures. Therefore, there are potential future research applications for astronauts to utilize nanomaterials that support bone-regeneration. For instance, in microgravity, the bone loss observed in astronauts resembles that of osteoporosis; nanostructured scaffolds could be tested in such conditions to counteract reduced osteogenesis and to validate their efficacy in extreme environments [282]. Moreover, nanomaterials could be used in wearable devices for bone health monitoring, targeted drug delivery, and fast injury healing to provide solutions for battling the environmental effects on the human biosystem in the ever-expanding space industry. Additionally, drugs can be designed in and for use in microgravity environments since microgravity boosts drug loading efficiency in nanocarriers and enhances protein crystal quality, both advanced therapeutics and functional coatings for aerospace applications can benefit to combat bone injury. (https://www.nasa.gov/missions/station/applications-within-reach/; accessed on 25 July 2025).

Gradually, the advancements in these domains will disclose new prospects and change the treatment strategies of BTE. Particularly, we foresee the evolution of new NMs and estimated barriers in the field of BTE intervention throughout the coming decades. The foundational progress and integration of artificial intelligence (AI)/machine learning (ML) in nanoscience will most likely replenish the current limitations; hence, the trajectories of NMs used in BTE will determine the new directions and precision increasingly in the field [283,284]. The current development is relying on refining smart stimuli-responsive materials to precisely deliver and stimulate the therapeutic agents to the osteogenic site. This approach will enable well-tuned systemic exposure and bone tissue regeneration. For instance, light-responsive NMs can enable the non-invasive and on-demand stimulation of the osteogenic processes. In addition, light responsiveness can aid the photothermal and photodynamic effects for attenuating unwanted bacterial growth. Similarly, refining NMs surface structure and properties with integrated pH, thermal, mechanical, and magnetic responsiveness will bring multiresolution for treating bone tissue injury through precise control over the microenvironment and therapeutic delivery [234]. Concurrently, formulating nanoscaffolds with the AI/ML-driven optimization and 3D bioprinting will allow biologically active, enhanced mechanical ability and patient-specific constructs for generating faster infusion and bone healing [236]. These 3D-printed nanoscaffolds with tailored porosity and microenvironment will enhance the ECM regeneration [285]. Based on our current understanding, nanorobotics could be another promising technology that can be used in in situ tissue printing directly inside the body, which will be less invasive/noninvasive compared to traditional surgery. For example, the nanorobot-driven biohybrid implants consisting of stem cells and NM-engineered exosomes may enable a dynamic release of essential cues to the targeted site. Furthermore, injectable bioink could open the dimension in BTR while self-assembling into a scaffold under the influence of magnetic resonance and nanorobots will repair microfractures. The programmable nanorobotics enables the detection of microfractures to immediately build mineral deposition. This technology can mimic the body’s own repair mechanism for creating ECM and achieving almost total defect repair within a short period of time. In addition, ML and AI technology can tell us the prospective materials and their immunocompetence while designing a nanoscaffold to result in nanomedicine and personalized precision medicine in bone repair. In addition, the ML/Al can predict the nature of new materials from the periodic table via investigating and finding the combination of biomaterials (metallic, organic, polymeric) for the synergistic interaction through analyzing the nanomaterial properties for thriving their tentative biological outcomes [286]. Thus, the algorithms can be used to analyze the patient-specific bone defect size, cell types, and nanomaterial composition for creating new personalized scaffolds to optimize the defect regeneration with less or no external and internal toxic outcomes. The nanomaterials—having bioelectric interfaces between conductive nanomaterials, along with the integration of ML/AI to monitor the osteoclast activities in real time—can pave the pathways toward preventing bone reabsorption. In a few decades, green nanomaterials will become a pivotal approach to attenuating the long-term environmental and toxicological threats. The inherent biocompatibility, biodegradability, high tunability, increased durability, affordability, scalability, and surface functionalization, once blended with the primary scaffold materials, will concretize a blueprint for sustainable nanomedicine for tissue regeneration; hence, the paradigm of green nanomaterials will overcome pitfalls from using the non-green counterparts.

Despite the thriving options, promising future potentials, and the ethical concerns and toxicity, research budgets—often far from providing the minimal need—might hinder new technological development. Therefore, overcoming the pitfalls, NMs in BTR will evolve into a key steppingstone in bone regenerative medicine.

## Figures and Tables

**Table 1:** Physical and functional implications of inorganic nanomaterials (calcium phosphate derivatives, oxide-based NMs) and polymers for refining bone tissue scaffold.

Materials		Average Pore Size (µm) of Individual Materials	Average Compressive Strength (MPa)-Porous	Average Elastic/Young’s Modulus (GPa)	Average Biodegradation Time (Month)	Average Scaffold Porosity (%)	References
Calcium Phosphate Derivatives	HAp	50–500	1–70	5–60	2–12	30–80	[38,39,40]
	TCP	100–500	1–70	1–20	3–20	30–75	[41,42,43,44]
	BCP	50–700	1–70	0.5–10	3–18	30–90	[38,45,46]
Oxide-Based NMs	GO	nanoporous	8.04–31.4 with HA	380–470 (inherent)	15 days (yellow mealworm)	80–99	[47,48,49,50]
	rGO	1–170	44–107 in composite matrices	250 (inherent)	Uncertain	50–95	[38,50,51]
	MgO	nanoporous	5–10	226–277 (dense)	1–9	50–99.7	[52,53,54,55]
	ZnO	nanoporous	3.01–18 with β–TCP; 146 with PCL	<1	Uncertain	56.8–87	[56,57,58]
	TiO_2_	mesoporous	15–25	<1	Non degradable	70–90	[59,60,61,62]
	SiO_2_	micro/macro/mesoporous	0.5–10	<1	1–3	70–95	[63,64]
	IOs	nanoporous	7.2–9.4	<1	1–12	45–80	[65,66,67]
Polymers and Polymer-Based NMs	PLGA	50–200	0.9–35	0.0094–0.51	0.4–24	4–90	[68,69,70]
	PLA	168	1–70	0.0016–0.071	6–24	70–90	[71,72]
	PGA	70–400	1–10	0.00012–0.00645	0.5–2	70–90	[73,74]
	PCL	100–500	1–77	0.0001–3.2	14–36	50–90	[75,76,77]

Note: The range shown in the table delineates the varying spectrums used and documented in previous research and indicates the variability of the specific characteristic in scaffold design.

## Data Availability

The data has been sourced from publicly accessible resources, primarily found within the PubMed and Scopus index databases. The designated resources along with their corresponding URLs are enumerated in the reference section.
